# Long-Term Management of Sympathetic Ophthalmia Developing in the Early Period Following Trauma: A Case Report

**DOI:** 10.7759/cureus.92281

**Published:** 2025-09-14

**Authors:** Rumeysa Bilmez Tan, Muhammed Batur, Veysi Yildiz

**Affiliations:** 1 Ophthalmology, SBU (Sağlık Bilimleri Üniversitesi) Van Training and Research Hospital, Van, TUR; 2 Ophthalmology, Urartu Eye Center, Van, TUR; 3 Ophthalmology, Van Yüzüncü Yıl University Faculty of Medicine, Van, TUR

**Keywords:** globe rupture, immunosuppressive therapy, sympathetic ophthalmia, traumatic eye injury, uveitis

## Abstract

This case report presents a rare instance of sympathetic ophthalmia (SO) in a patient who developed globe rupture due to blunt ocular trauma. It provides insights into the clinical course and three-year follow-up of successful treatment achieved through systemic immunosuppressive therapy. A 45-year-old male patient sustained blunt trauma to his left eye. Due to clinical signs indicative of rupture, conjunctival exploration was performed, revealing a scleral rupture that was primarily repaired. On postoperative day 18, the patient developed bilateral panuveitis consistent with trauma-induced SO. Diagnosis was supported by clinical signs, optical coherence tomography (OCT), and fundus fluorescein angiography (FFA). Treatment included systemic corticosteroids and immunosuppressive agents (cyclosporine and azathioprine), with dose adjustments during follow-up. Three years later, following cataract surgery, visual acuity in the injured eye improved to 0.7 (Snellen). OCT and FFA findings normalized, and systemic therapy was well tolerated except for mild gingival hyperplasia attributed to cyclosporine. This case underscores the importance of early diagnosis and aggressive immunosuppressive therapy in managing early-onset SO following globe rupture. Long-term follow-up demonstrated effective inflammation control and stable visual acuity, supporting the efficacy of combined immunosuppressive therapy.

## Introduction

Sympathetic ophthalmia (SO) is a variable form of uveitis that develops following penetrating or, more rarely, non-penetrating ocular trauma [1]. Originally described in 1840 by William Mackenzie, as cited by Albert and Diaz-Rohena [2], SO remains a rare but serious complication that can lead to bilateral visual impairment if not promptly diagnosed and treated. The reported incidence of SO after open globe injuries ranges from 0.1% to 3% [3]. A comprehensive meta-analysis evaluating 24 population-based studies reported an incidence of 0.19% [3], while another meta-analysis found an incidence of 0.12% [4]. Despite these relatively low incidence rates, the potential for bilateral visual loss makes SO a condition of signiﬁcant clinical concern.

Despite its long history in ophthalmic literature, the exact etiology and pathophysiology of SO remain incompletely understood. Most contemporary studies support an autoimmune origin for this condition. The most widely accepted theory suggests that ocular antigens, normally isolated from the immune system by the blood-retinal barrier, are released due to disruption of this barrier caused by trauma or surgery [3,5]. This exposure leads to a T-cell-mediated autoimmune reaction that can aﬀect both the injured and the fellow eye [1,6].

SO can have an acute or insidious onset and may occur in days to several years after the inciting event. The temporal range of onset is remarkably broad, with cases reported as early as ﬁve days and as late as 66 years post trauma [7,8]. Most cases manifest within the ﬁrst year and typically follow a relapsing-remitting pattern [3]. SO generally presents as posterior uveitis that may progress to panuveitis, aﬀecting one or both eyes [1,9].

Diagnosis is based on patient history and clinical features of intraocular inﬂammation. Ancillary investigations, including optical coherence tomography (OCT) and fundus ﬂuorescein angiography (FFA), support the diagnosis, assess disease severity, and help monitor treatment response [10]. The cornerstone of treatment is systemic corticosteroids, initially to control inﬂammation, followed by steroid-sparing immunosuppressive agents [3].

In this case report, we present the long-term management of a patient diagnosed with SO on the 18th day following globe rupture, highlighting the clinical course and successful therapeutic response over a three-year follow-up period. This case demonstrates the importance of early recognition and aggressive immunosuppressive therapy in achieving favorable visual outcomes.

## Case presentation

**Figure 1:**
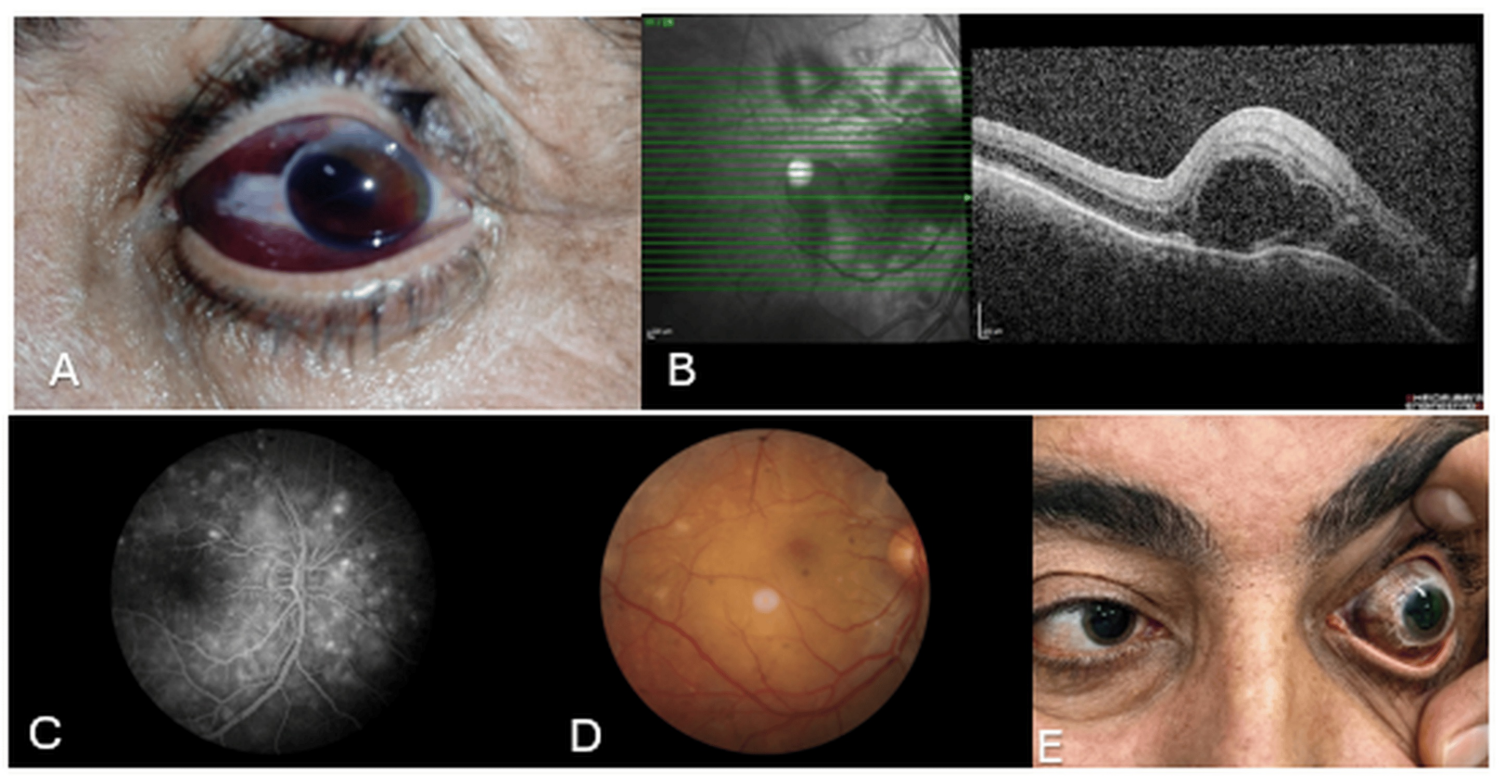
Clinical presentation and multimodal imaging findings in sympathetic ophthalmia following traumatic globe rupture. (A) Photograph of the left eye immediately after trauma showing subconjunctival hemorrhage, shallow anterior chamber with grade 1 hyphema, and distorted pupil superonasally, consistent with globe rupture. (B) Optical coherence tomography of the right eye on postoperative day 18 demonstrating multiple subretinal and intraretinal fluid spaces, septa between the spaces, elongation of the outer photoreceptor segments, increased choroidal thickness, and disrupted choroidal architecture characteristic of sympathetic ophthalmia. (C, D) Fundus fluorescein angiography images showing multiple pinpoint hyperfluorescent leakages in the arteriovenous phase and Dalen-Fuchs nodules, supporting the diagnosis of sympathetic ophthalmia. (E) Clinical photograph demonstrating widespread conjunctival melanosis in the left eye at week 19 postoperatively, a characteristic finding in sympathetic ophthalmia.

A 45-year-old male patient experienced blunt trauma to his left eye caused by an impact from pliers. Initial examination revealed visual acuity of 20/20 in the right eye and light perception in the left eye. Slit-lamp examination of the right eye showed normal anterior and posterior segments, while the left eye demonstrated subconjunctival hemorrhage inferiorly and temporally. The anterior chamber was shallow with grade 1 hyphema. The pupil was distorted superonasally, and the intraocular pressure was hypotonic in the left eye (Figure 1A). Based on clinical findings suggestive of globe rupture, conjunctival exploration was performed under general anesthesia.

Surgical exploration revealed a 10 mm scleral rupture in the superotemporal quadrant (zone 2), which was repaired with 8-0 Vicryl sutures. Postoperatively, the patient received systemic antibiotic therapy with cefazolin and gentamicin, systemic prednisolone (1 mg/kg), a proton pump inhibitor, and intensive topical therapy including fortified cefazolin and gentamicin drops, prednisolone drops hourly, and cycloplegic drops three times daily.

On postoperative day 1, visual acuity in the left eye improved to counting fingers from 20 cm. Slit-lamp examination revealed corneal edema, Descemet's folds, and a pupillary membrane. Fundus examination showed vitreous hemorrhage and retinal hemorrhage near the inferior vascular arcade. B-scan ultrasonography confirmed an attached retina with hyperreflective densities consistent with vitreous hemorrhage. Systemic treatment was discontinued after one week, and the patient was discharged with continued topical therapy.

By postoperative day 14, visual acuity was 20/20 (Snellen) in the right eye and counting fingers at 1.5 meters in the left eye. The right anterior and posterior segments remained normal. In the left eye, there was residual subconjunctival hemorrhage, partial traumatic aniridia, and early posterior subcapsular cataract formation. The retina appeared normal with residual vitreous hemorrhage. Intraocular pressure was measured as 14 mmHg in the right eye and 12 mmHg in the left eye.

On postoperative day 18, the patient presented with bilateral visual deterioration. Visual acuity had decreased to 20/67 (Snellen) in the right eye and to hand motion in the left eye. The anterior segment of the right eye was quiet, but fundus examination revealed multiple serous retinal detachments. The anterior segment of the left eye was also quiet with persistent vitreous hemorrhage. OCT of the right eye showed multiple subretinal and intraretinal fluid spaces, septa between the spaces, elongation of the outer photoreceptor segments, increased choroidal thickness, and disrupted choroidal architecture (Figure 1B). OCT images of the left eye were obscured due to persistent hemorrhage. FFA revealed multiple pinpoint hyperfluorescent leakages in the arteriovenous phase and Dalen-Fuchs nodules (Figures 1C, 1D). These findings were consistent with a diagnosis of SO.

Laboratory work-up, including complete blood count, comprehensive metabolic panel (liver enzymes, kidney function tests, serum electrolytes), and enzyme-linked immunoassay (ELISA) panels (hepatitis B virus (HBV), hepatitis C virus (HCV), HIV, and TORCH (toxoplasmosis, rubella, cytomegalovirus, herpes simplex, and syphilis) screening), were within normal limits. Treatment was initiated with intravenous prednisolone 1 g/day for five days, oral cyclosporine 100 mg twice daily, and hourly topical prednisolone drops. As the QuantiFERON-TB Gold test (QIAGEN, Hilden, Germany) was positive, a full work-up for active tuberculosis (TB) was performed, including chest X-ray and clinical assessment, which were unremarkable. Based on the absence of clinical or radiologic evidence of active disease, isoniazid monotherapy was initiated as prophylaxis at a dose of 300 mg/day in accordance with standard latent TB treatment protocols. The systemic steroid dose was tapered from 2 mg/kg to 1 mg/kg and then gradually reduced over several weeks.

At week 16 postoperatively, visual acuity had improved to 20/20 (Snellen) in the right eye and 20/133 (Snellen) in the left eye. Anterior chambers were quiet bilaterally. Fundus examination showed no signs of active inflammation. OCT revealed resolution of subretinal fluid. As inflammation was under control, systemic prednisolone was discontinued while cyclosporine 100 mg twice daily and topical prednisolone hourly were continued.

At week 19 postoperatively, the patient developed recurrent subretinal and intraretinal fluid in the right eye, along with widespread conjunctival melanosis in the left eye (Figure 1E). Azathioprine 50 mg twice daily was added to the regimen, and oral prednisolone 1 mg/kg was restarted and tapered within one month.

**Figure 2:**
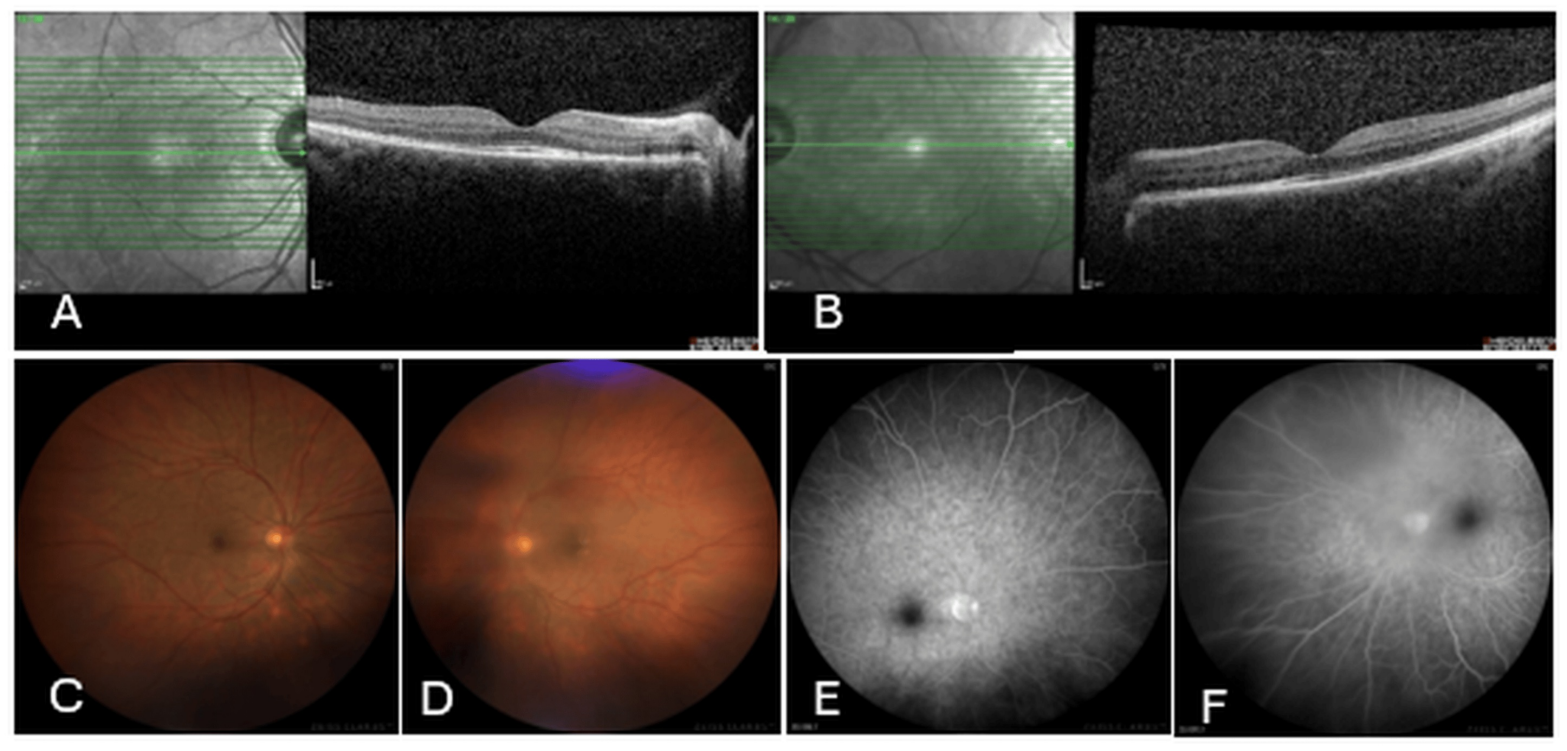
Multimodal imaging at the one-year follow-up: optical coherence tomography (OCT), fundus photograph, and fundus fluorescein angiography (FFA) (A, B) OCT images at one-year follow-up showing resolution of subretinal fluid bilaterally, with some areas of inner segment/outer segment (IS/OS) disruption noted in the right macula. (C-F) Fundus photograph and FFA images at one-year follow-up demonstrating no active leakage, indicating successful control of inflammation with immunosuppressive therapy.

At the one-year follow-up, visual acuity was 20/20 (Snellen) in the right eye and 20/67 (Snellen) in the left eye. The right anterior segment was normal. In the right fundus, mild retinal pigment epithelium (RPE) changes were observed, with no signs of Dalen-Fuchs nodules or serous detachment. In the left eye, there was conjunctival melanosis, partial traumatic aniridia, and posterior subcapsular cataract in the anterior segment. Inactive vitritis was present in the posterior segment, but no signs of retinitis or retinal detachment were noted. OCT showed no subretinal fluid bilaterally, though inner segment/outer segment (IS/OS) disruption was noted in some areas of the right macula (Figures 2A, 2B). FFA showed no active leakage (Figures 2C-2F). Systemic treatment with cyclosporine and azathioprine was continued. Mild gingival hyperplasia was observed as a side effect of cyclosporine therapy. Cataract surgery was performed on the left eye with phacoemulsification and in-the-bag monofocal intraocular lens implantation. At the third postoperative year, visual acuity was 20/20 (Snellen) in the right eye and 20/29 (Snellen) in the left eye, with no recurrence of inflammation during the follow-up period.

**Figure 3:**
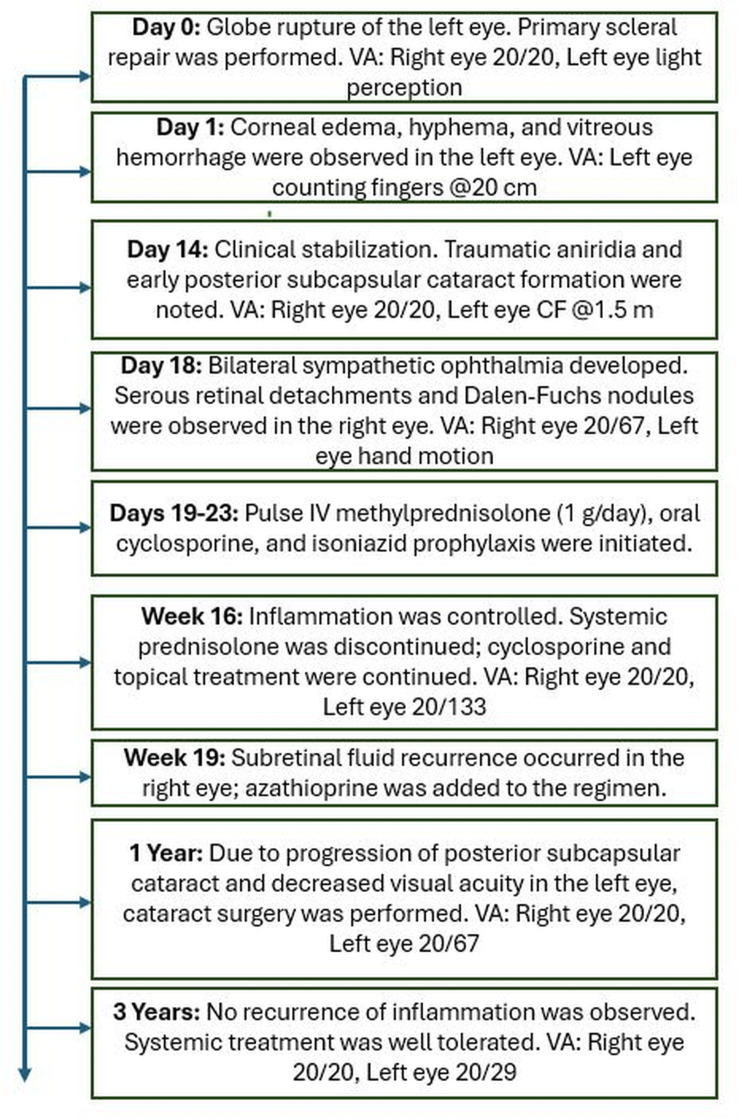
Timeline of clinical events in a patient with bilateral sympathetic ophthalmia. The patient experienced blunt trauma to the left eye, followed by globe rupture, surgical repair, inflammatory complications, and systemic treatment. Visual acuity and treatment modifications are shown for each major clinical stage. VA: visual acuity; CF: count fingers

The chronological progression of clinical findings, interventions, and treatment outcomes is summarized in Figure 3.

## Discussion

In our patient, SO was successfully controlled and treated with appropriate medical therapy. Although histopathological examination was not performed, the widespread conjunctival melanosis observed during follow-up supports the widely accepted hypothesis that SO arises due to exposure of previously sequestered ocular antigens following trauma or surgery that disrupts the blood-retinal barrier [3].

Historically, it was believed that severely traumatized eyes with no visual potential should be enucleated within the first two weeks to prevent SO. However, this approach is now controversial [11]. While early enucleation was thought to reduce risk, cases of SO have still been reported even after removal of the inciting eye [12]. According to current consensus, enucleation is recommended only if globe repair is not feasible and there is no visual potential; otherwise, prophylactic eye removal is not advised [13].

Systemic corticosteroids remain the cornerstone of SO treatment. The standard protocol begins with high-dose intravenous methylprednisolone followed by oral prednisolone, which is tapered over a period of up to six months. Topical and periocular corticosteroids may be used as adjuncts but can lead to serious ocular complications. Due to systemic side effects of corticosteroids, combining them with immunosuppressive agents is recommended. Multiple immunosuppressive combinations have been shown to be effective in refractory cases [14]. Modern treatment approaches have also led to a reduction in recurrence rates compared to earlier periods.

In refractory SO cases, tumor necrosis factor-alpha (TNF-α) and interleukin (IL)-6 inhibitors such as adalimumab, infliximab, and tocilizumab have been successfully used. Future therapeutic strategies targeting Th17/IL-23 pathways, macrophage activation, and chemokine signaling are considered promising [14]. Ganesh et al. reported that in resistant cases, a combination of prednisolone, azathioprine, and cyclosporine might be necessary [15]. In our case, we also achieved remission using this combination. Considering the recurrence observed under intensive corticosteroid therapy, earlier introduction of a TNF inhibitor might have been beneficial. Notably, adalimumab, a TNF inhibitor, has been recommended for refractory cases, and some studies suggest that early initiation may accelerate inflammation control [14].

In a study by Kilmartin et al., 75% of patients diagnosed with SO achieved a visual acuity of 6/12 or better within one year after systemic immunosuppressive therapy. These findings call into question the necessity of prophylactic eye removal [16]. Our case supports these findings, as the patient achieved excellent visual outcomes in both eyes with appropriate medical management.

SO should be considered in cases of uveitis developing after ocular trauma. The diagnosis can be supported in the early phase using multimodal imaging, including OCT and FFA, and inflammation can be effectively controlled with timely systemic therapy. Prior to initiating immunosuppressive therapy, patients should be evaluated for latent TB activation. For this purpose, purified protein derivative (PPD) or preferably a QuantiFERON test should be performed, and prophylaxis initiated when necessary.

In our case, the diagnosis was made in the third week post trauma, and treatment with systemic corticosteroids and immunosuppressive agents was initiated promptly. During the three-year follow-up, stable visual acuity of 20/20 (Snellen) in the right eye and 20/29 (Snellen) in the left eye was maintained. This outcome demonstrates that timely and appropriate modern immunosuppressive treatment can lead to successful visual outcomes without resorting to enucleation.

Had the injured eye been enucleated early in this case, a visually functional eye with the potential to reach 20/29 (Snellen) visual acuity would have been lost. Therefore, enucleation should only be considered when globe repair is not possible and no visual potential exists. This case emphasizes the importance of early recognition, prompt treatment, and long-term follow-up in managing SO following traumatic globe rupture.

## Conclusions

This case demonstrates a rare early-onset presentation of SO occurring within days after trauma, emphasizing the critical importance of prompt diagnosis and aggressive immunosuppressive therapy. With timely surgical and medical intervention, favorable long-term visual outcomes were achieved in both eyes without the need for enucleation. The emergence of new visual symptoms in the fellow eye should raise immediate clinical suspicion, prompting swift evaluation and treatment to preserve vision. This case further highlights the potential for late complications such as recurrent inflammation and cataract progression, underlining the necessity of long-term follow-up and treatment adjustments. The requirement for multiple immunosuppressive agents during management reflects the complexity and challenges of clinical decision-making in such cases. Lastly, this report reinforces the importance of globe-preserving strategies; had primary enucleation been pursued, a potentially salvageable eye would have been lost. Therefore, unless the globe is irreparably damaged, a conservative approach should be prioritized.
